# Influence of CYP2C19 Metabolizer Status on Escitalopram/Citalopram Tolerability and Response in Youth With Anxiety and Depressive Disorders

**DOI:** 10.3389/fphar.2019.00099

**Published:** 2019-02-19

**Authors:** Stacey L. Aldrich, Ethan A. Poweleit, Cynthia A. Prows, Lisa J. Martin, Jeffrey R. Strawn, Laura B. Ramsey

**Affiliations:** ^1^Department of Pediatrics, University of Cincinnati College of Medicine, Cincinnati, OH, United States; ^2^Division of Human Genetics, Cincinnati Children’s Hospital Medical Center, Cincinnati, OH, United States; ^3^Division of Research in Patient Services, Cincinnati Children’s Hospital Medical Center, Cincinnati, OH, United States; ^4^Division of Patient Services, Cincinnati Children’s Hospital Medical Center, Cincinnati, OH, United States; ^5^Anxiety Disorders Research Program, Department of Psychiatry and Behavioral Neuroscience, University of Cincinnati, Cincinnati, OH, United States; ^6^Division of Child and Adolescent Psychiatry, Cincinnati Children’s Hospital Medical Center, Cincinnati, OH, United States

**Keywords:** pharmacogenetics, CYP2C19, SSRI (selective serotonin reuptake inhibitor), antidepressant, anxiety disorders, depressive disorder, escitalopram, citalopram

## Abstract

In pediatric patients, the selective serotonin reuptake inhibitors (SSRIs) escitalopram and citalopram (es/citalopram) are commonly prescribed for anxiety and depressive disorders. However, pharmacogenetic studies examining CYP2C19 metabolizer status and es/citalopram treatment outcomes have largely focused on adults. We report a retrospective study of electronic medical record data from 263 youth < 19 years of age with anxiety and/or depressive disorders prescribed escitalopram or citalopram who underwent routine clinical *CYP2C19* genotyping. Slower CYP2C19 metabolizers experienced more untoward effects than faster metabolizers (*p =* 0.015), including activation symptoms (*p* = 0.029) and had more rapid weight gain (*p* = 0.018). A larger proportion of slower metabolizers discontinued treatment with es/citalopram than normal metabolizers (*p* = 0.007). Meanwhile, faster metabolizers responded more quickly to es/citalopram (*p* = 0.005) and trended toward less time spent in subsequent hospitalizations (*p* = 0.06). These results highlight a disparity in treatment outcomes with es/citalopram treatment in youth with anxiety and/or depressive disorders when standardized dosing strategies were used without consideration of CYP2C19 metabolizer status. Larger, prospective trials are warranted to assess whether tailored dosing of es/citalopram based on CYP2C19 metabolizer status improves treatment outcomes in this patient population.

## Introduction

Up to 10% of children and adolescents in the United States may have an anxiety or depressive disorder (Merikangas et al., 2010). Additionally, psychiatric disorders are among the most expensive conditions to treat in pediatric patients (Soni, 2015) and approximately 1 in 10 pediatric hospitalizations is related to a primary psychiatric disorder (Bardach et al., 2014). These disorders are commonly treated with selective serotonin reuptake inhibitors (SSRIs); however, treatment response is variable. Myriad factors, including genetics, underlie this variability in treatment response to SSRIs. As such, pharmacogenomics studies of SSRI-treated youth may help to optimize outcomes in SSRI-treated youth by providing personalized medication and dosing recommendations based on genotypes (Wehry et al., 2018).

In pediatric patients with anxiety and depressive disorders, SSRIs decrease symptoms, restore functioning and improve quality of life (Strawn et al., 2015, 2018; Cipriani et al., 2016; Locher et al., 2017; Dobson et al., 2019). Escitalopram and citalopram (es/citalopram) are among the most frequently prescribed antidepressants in pediatric patients (Czaja et al., 2013), yet approximately 50% of these patients fail to respond (Wagner et al., 2004, 2006; Goodman et al., 2005; Isolan et al., 2007; Emslie et al., 2009). Side effects are common, with about 5% of patients experiencing intolerable side effects that lead them to discontinue treatment (Wagner et al., 2004, 2006; Goodman et al., 2005; Isolan et al., 2007; Emslie et al., 2009).

Es/citalopram, like other SSRIs, prolong the availability of synaptic serotonin (5-hydroxytryptamine, 5-HT) (Tatsumi et al., 1997). Escitalopram is composed entirely of the therapeutically active S-enantiomer, while citalopram is a racemic mixture of both the S-enantiomer and therapeutically inactive R-enantiomer (LEXAPRO, 2018). Es/citalopram is primarily metabolized by the hepatic CYP2C19 enzyme (von Moltke et al., 1999; Herrlin et al., 2003; Huezo-Diaz et al., 2012). Alleles that contain variants in the *CYP2C19* gene – categorized as no function, normal function or increased function – directly modulate the enzyme’s efficiency in es/citalopram metabolism (Hicks et al., 2015). The metabolizer status is determined by the two alleles a person carries, into poor, intermediate, normal, rapid or ultrarapid metabolizer (UM) status (Caudle et al., 2017).

The efficacy and tolerability of es/citalopram has been extensively evaluated in the pediatric population (Wagner et al., 2004, 2006; Findling et al., 2006, 2013; Isolan et al., 2007; Emslie et al., 2009), but pharmacogenetic studies are lacking. In adults, faster CYP2C19 metabolizers have lower serum es/citalopram concentrations at equivalent doses, compared with normal metabolizers (NMs), while slower CYP2C19 metabolizers have increased serum concentrations (Altar et al., 2013; Chang et al., 2014; Jukic et al., 2018). The influence of CYP2C19 metabolizer status on plasma concentration does not differ for escitalopram and citalopram (Chang et al., 2014). Therefore, faster CYP2C19 metabolizers may be at greater risk for treatment failure, and slower CYP2C19 metabolizers may experience more side effects when treated with these medications (Hicks et al., 2015). Notably, age is also associated with es/citalopram exposure in adults, with older individuals demonstrating higher serum concentrations relative to younger adults (Jin et al., 2010; Huezo-Diaz et al., 2012; Jukic et al., 2018). However, the impact of CYP2C19 metabolizer status on serum concentrations of es/citalopram in pediatric patients is largely unknown (Jackson, 2008; Strawn et al., 2019), while studies investigating *CYP2C19* genotype and treatment outcomes with es/citalopram are largely restricted to adults (Mrazek et al., 2011; Hodgson et al., 2014).

The Clinical Pharmacogenetics Implementation Consortium (CPIC) guidelines for es/citalopram dosing based on CYP2C19 metabolizer status (Hicks et al., 2015) advise that clinicians should consider alternative medications that are not predominantly metabolized by CYP2C19 in poor and ultrarapid CYP2C19 metabolizers. However, CPIC warns its guidelines should be used with caution in children, citing the lack of research in pediatric populations and the fact that CYP2C19 activity may be increased in children relative to adults. However, the studies of the ontogeny of *CYP2C19* show equivalent expression after the age of 1 through adulthood (Koukouritaki et al., 2004), although they do not take into account how the ^∗^17 allele influences expression (Sanford et al., 2013). The studies that demonstrate increased clearance in adolescents compared to adults do not take into account *CYP2C19* genetic variants that influence expression, so this difference could be due to a larger proportion of faster metabolizers being included in the adolescent cohort than the adult cohort.

In light of this information gap, we retrospectively analyzed electronic medical record (EMR) data to investigate the association between CYP2C19 metabolizer status and treatment outcomes following inpatient psychiatric hospitalization in youth with anxiety and/or depressive disorders. We hypothesized that slower CYP2C19 metabolizers would experience more side effects and higher response rates compared to faster CYP2C19 metabolizers, based on exposure trends seen in adults (Chang et al., 2014; Jukic et al., 2018).

## Materials and Methods

### Subjects

A query was developed to identify potentially eligible patients in the EMR who were admitted to the inpatient psychiatric unit at Cincinnati Children’s Hospital Medical Center (CCHMC) between January 2010 and May 2017. Inclusion criteria were as follows: a new prescription of es/citalopram initiated at <19 years old; a diagnosed anxiety and/or depressive disorder; and *CYP2C19* genotyping performed after September 1, 2013, when we began testing patients for an expanded set of allele compared to prior testing (Ramsey et al., 2018b). Exclusion criteria were as follows: a thyroid stimulating hormone level of >5.5 mIU/L as reviewed by a board-certified physician (JRS), or a diagnosis of traumatic brain injury, substance use disorder, intellectual disability, congenital brain abnormality and/or bipolar disorder. The total treatment period with es/citalopram was the number of consecutive days between the prescription start date and end date. Overlapping prescriptions with 25 psychotropic medications during the es/citalopram treatment period were assessed (bupropion, desvenlafaxine, duloxetine, fluoxetine, fluvoxamine, mirtazapine, sertraline, venlafaxine, aripiprazole, asenapine, lurasidone, olanzapine, paliperidone, prochlorperazine, quetiapine, risperidone, ziprasidone, alprazolam, buspirone, clobazam, clonazepam, clonidine, guanfacine, hydroxyzine and lorazepam). All data were abstracted from the patients’ EMR and the reviewer was blind to CYP2C19 metabolizer status during data abstraction. The study protocol was approved by the Institutional Review Board at CCHMC and determined to be no more than minimal risk to the patients according to the US Department of Health and Human Services Office for Human Research Protections policies 45 CFR 46.110 and 21 CFR 56.110.

### Side Effects

To be considered for the tolerability cohort, patients must have been prescribed es/citalopram for a minimum of one day and had at least one psychiatric encounter note recorded in their EMR during the total treatment period with es/citalopram. We assessed ten side effects previously reported in pediatric patients taking es/citalopram: activation, drowsiness, gastrointestinal symptoms, headache, hyperactivity, impulsivity, insomnia, irritability, nausea and weight gain (Luft et al., 2018). We applied an adaptive natural language process to develop an algorithm that looked for the presence of key side effect-related terms in more than 32,000 EMR notes. To do this, we first performed a manual review of the EMR notes to identify common words and phrases that providers used to document the presence of these side effects. We applied this algorithm to scan each psychiatry encounter note during es/citalopram treatment. We then manually reviewed charts to refine the algorithm and to achieve a false-positive rate <10% for each side effect assessed. For example, irritability was coded as positive with the presence of “agitation,” “agitated,” “irritable,” “irritability” and/or “irritated,” but phrases such as “no irritability,” “not irritable” or “irritable bowel syndrome” were ignored. The algorithm also assessed for adherence concerns in the EMR notes. Adherence was assessed by the treating clinician using a 0–4 numerical scale (0 = no concerns; 1 = mild concerns; 2 = moderate concerns; 3 = severe concerns; and 4 = very severe concerns). Non-adherence was defined as the presence of a 1, 2, 3 or 4. Time to first side effect was the length in days between the prescription start date and the earliest date when one of the side effect-related terms appeared in the EMR notes during the total treatment period with es/citalopram. The sum of side effects was the cumulative number of positive side effects during the es/citalopram treatment period. Total inpatient days (hospitalizations after the initial hospitalization where *CYP2C19* testing was performed) was the total number of days spent in the inpatient psychiatric unit during the total treatment period with es/citalopram, excluding the initial inpatient visit (when es/citalopram was often prescribed).

### Response

To be included in the response cohort, patients must have been prescribed es/citalopram for a minimum of 28 consecutive days and had at least one Clinical Global Impression-Improvement (CGI-I) score (Guy, 1976) recorded by a clinician during the es/citalopram treatment period. The CGI-I score has been used in previous clinical trials of es/citalopram to assess treatment response in pediatric patients and is used routinely in our institution (Baumgartner et al., 2002; Prince et al., 2002; Wagner et al., 2004, 2006; Goodman et al., 2005; Isolan et al., 2007; Emslie et al., 2009; Schirman et al., 2010; Findling et al., 2013). Responders were defined as patients who achieved at least one CGI-I score of 1 (“very much improved”) or 2 (“much improved”) on a scale of 1–7 during the total es/citalopram treatment period. All other patients were categorized as non-responders. The response dose was the dosage of es/citalopram in milligrams per day prescribed seven days prior to the first CGI-I score of 1 or 2. The maximum dose was the highest dose of es/citalopram in milligrams per day prescribed during the total treatment period with es/citalopram. Citalopram dosage was standardized to escitalopram in a 2:1 ratio (Owens et al., 2001). Patients may have been treated with both medications (switched from one to the other), in this case, the highest dose of either medication was taken as the highest dose. Time to response was the number of days between the prescription start date and when the first CGI-I score of 1 or 2 was recorded. Time to response dose was the number of days between the prescription start date and the first date the response dose was prescribed.

### *CYP2C19* Genotyping

*CYP2C19* genotyping is routinely performed on all patients admitted to the CCHMC psychiatric unit through the institution’s Molecular Genetics Laboratory (which has certification from the College of American Pathologists and Clinical Laboratory Improvement Amendments) (Ramsey et al., 2018b). Genomic DNA was isolated from peripheral blood or buccal swabs using the MagNA Pure Compact System (Roche Applied Science, Indianapolis, IN) and quantified by the NanoDrop Spectrophotometer (Thermo Fisher Scientific, Wilmington, DE). *CYP2C19* genotype was determined using the TaqMan allelic discrimination system (Life Technology, Forest City, CA) on a low density microarray. The assay detected seven no function alleles (*^∗^2, ^∗^3, ^∗^4, ^∗^5, ^∗^6, ^∗^7* and *^∗^8*) and the increased function allele (*^∗^17*). The *^∗^1* genotype was inferred from the absence of the preceding alleles. CPIC guidelines were used to categorize patients as poor, intermediate, normal, rapid or ultrarapid CYP2C19 metabolizers (Hicks et al., 2015; Moriyama et al., 2016; Caudle et al., 2017). Poor metabolizers (PMs) had two no function alleles. Intermediate metabolizers (IMs) had one no function allele and one normal function allele or increased function allele. NMs had two normal function alleles (^∗^1/^∗^1). Rapid metabolizers (RMs) had one normal function allele and one increased function allele (^∗^1/^∗^17). UMs had two increased function alleles (^∗^17/^∗^17). A report with the patient’s metabolizer status for CYP2C19 and CYP2D6 is included in the EMR with dosing recommendations for 19 neuropsychiatric medications, not including citalopram or escitalopram. Clinical decision support exists for the 19 medications included on the report (Ramsey et al., 2018b).

### Statistics

Metabolizer status was treated as an ordinal variable from PM to UM. ANOVA test for trend was used to test association of CYP2C19 with outcomes. Binary variables were analyzed with general linear models and continuous variables were analyzed with linear models in *R* version 3.2.2 ^1^. CYP2C19 activity is inhibited by some medications, including oral contraceptives (Carlsson et al., 2001; Reis et al., 2007) and proton pump inhibitors (Rocha et al., 2010; Gjestad et al., 2015). When analyzing associations of CYP2C19 metabolizer status with outcomes, concomitant use of oral contraceptives and the proton pump inhibitor omeprazole were included in the models. Time to response was analyzed with the log-rank test for trend using Prism 7 for Windows (GraphPad, La Jolla, CA). For secondary analyses, pairwise comparisons with null testing for correlation were used. Discontinuation of medication was analyzed with the Chi-square test for trend using Prism. *p*-values of <0.05 were considered statistically significant.

## Results

Of the 263 potential patients identified by the EMR query, 248 met inclusion criteria for the tolerability cohort (Table 1) and 180 met inclusion criteria for the response cohort (Table 2), and the overlap between the two cohorts was 170 patients. The majority of the patients received escitalopram, with a small number of patients receiving citalopram or switching from one to the other (Tables 1, 2). Inclusion and exclusion criteria are detailed in the Methods section. With regards to es/citalopram treatment time, patients included in the tolerability cohort must have received the medication at least one day, while in the response cohort patients must have received the medication for at least 28 days.

**Table 1:** Demographic and prescription data of tolerability cohort by CYP2C19 phenotype.

Parameter	Total *n* = 248	PM *n* = 5 (2%)	IM *n* = 57 (23%)	NM *n* = 100 (40%)	RM *n* = 73 (29%)	UM *n* = 13 (5%)
**Age (years)** Average (range)	14.4 (6.4–18.8)	13.9 (12.1–15.3)	14.4 (6.8–18.9)	14.6 (7.8–18.5)	14.2 (6.4–18.6)	14.8 (12.0–17.7)
<12 (*n*, %)	27 (11%)	–	7 (12%)	8 (8%)	12 (16%)	–
≥12 (*n*, %)	221 (89%)	5 (100%)	50 (88%)	92 (92%)	61 (84)	13 (100%)
**Diagnosis**						
Anxiety (*n*, %)	30 (12%)	1 (20%)	5 (9%)	13 (13%)	11 (15%)	–
Depressive (*n*, %)	68 (27%)	–	19 (33%)	25 (25%)	16 (22%)	8 (62%)
Anxiety+Depressive (*n*, %)	150 (61%)	4 (80%)	33 (58%)	62 (62%)	46 (63%)	5 (38%)
**Sex**						
Female (*n*, %)	162 (65%)	3 (60%)	41 (72%)	63 (63%)	47 (64%)	8 (62%)
Male (*n*, %)	86 (35%)	2 (40%)	16 (28%)	37 (37%)	26 (36%)	5 (38%)
**Race**						
Black (*n*, %)	23 (9%)	0 (0%)	7 (12%)	7 (7%)	7 (10%)	2 (15%)
Other (*n*, %)	25 (10%)	2 (40%)	7 (12%)	11 (11%)	4 (5%)	1 (8%)
White (*n*, %)	200 (81%)	3 (60%)	43 (76%)	82 (82%)	62 (85%)	10 (77%)
**Es/citalopram prescriptions** *n* (%)						
Only escitalopram	197 (79%)	4 (80%)	46 (81%)	79 (79%)	56 (77%)	12 (92%)
Only citalopram	38 (15%)	1 (20%)	6 (11%)	16 (16%)	14 (19%)	1 (8%)
Both	13 (5%)	0	5 (9%)	5 (5%)	3 (4%)	0
**Total time on es/citalopram (days)** Median (range)	236 (1–1974)	137 (35–453)	240 (1–1974)	271.5 (5–1424)	231 (1–1589)	171 (20–462)
**# of concomitant medications** Median (range)	2 (0–11)	2 (1–5)	2 (0–11)	2 (0–11)	1 (0–8)	2 (0–4)
**Concomitant omeprazole** *n* (%)	22 (9%)	1 (20%)	5 (9%)	6 (6%)	7 (10%)	3 (30%)
**Concomitant oral contraceptives** *n* (%)	24 (10%)	0 (0%)	7 (12%)	12 (12%)	4 (5%)	1 (8%)
**Adherence concerns** (*n*, %)	26 (10%)	0 (0%)	8 (14%)	12 (12%)	3 (4%)	3 (23%)
**Maximum dose (mg)** Median (range)	15 (2.5–40)	15 (5–35)	15 (2.5–40)	17.5 (2.5–40)	15 (2.5–40)	20 (5–20)


*n, number; mg, milligrams; PM, poor metabolizer; IM, intermediate metabolizer; NM, normal metabolizer; RM, rapid metabolizer; UM, ultrarapid metabolizer.*

**Table 2:** Demographic and prescription data of response cohort by CYP2C19 phenotype.

Parameter	Total *n* = 180	PM *n* = 4 (2%)	IM *n* = 41 (23%)	NM *n* = 77 (43%)	RM *n* = 48 (26%)	UM *n* = 10 (6%)
**Age (years)** Average (range)	14.8 (6.8–18.6)	13.5 (12.1–15.2)	14.9 (6.8–17.5)	14.8 (9.7–18.6)	14.6 (9.4–18.6)	15.2 (12.0–17.4)
<12 (*n*, %)	18 (10%)	–	5 (12%)	7 (9%)	6 (13%)	–
≥12 (*n*, %)	162 (90%)	4 (100%)	36 (88%)	70 (91%)	42 (87%)	10 (100%)
**Diagnosis**						
Anxiety (*n*, %)	22 (12%)	1 (25%)	5 (12%)	10 (13%)	6 (12%)	–
Depressive (*n*, %)	44 (25%)	0	12 (29%)	20 (26%)	9 (19%)	3 (30%)
Anxiety+Depressive (*n*, %)	114 (63%)	3 (75%)	24 (59%)	47 (61%)	33 (69%)	7 (70%)
**Sex**						
Female (*n*, %)	119 (66%)	2 (50%)	29 (71%)	49 (64%)	33 (69%)	6 (60%)
Male (*n*, %)	61 (34%)	2 (50%)	12 (29%)	28 (36%)	15 (31%)	4 (40%)
**Race**						
Black (*n*, %)	16 (9%)	0	5 (12%)	5 (7%)	4 (8%)	2 (20%)
Other (*n*, %)	18 (10%)	1 (25%)	6 (15%)	8 (10%)	3 (6%)	–
White (*n*, %)	146 (81%)	3 (75%)	30 (73%)	64 (83%)	41 (86%)	8 (80%)
**Es/citalopram prescriptions** *n* (%)						
Only escitalopram	147 (82%)	3 (75%)	46 (88%)	60 (78%)	39 (81%)	9 (90%)
Only citalopram	24 (13%)	1 (25%)	3 (7%)	12 (16%)	7 (15%)	1 (10%)
Both	9 (5%)	0	2 (5%)	5 (6%)	2 (4%)	0
**Total time on es/citalopram (days)** Median (range)	290.5 (28–1974)	112 (35–293)	284 (30–1974)	328 (31–1424)	290 (28–1206)	188 (32–1129)
**Response** *n* (%)	117 (65%)	1 (25%)	23 (56%)	49 (64%)	37 (77%)	7 (70%)
**# of concomitant medications** Median (range)	2 (0–9)	1.5 (1–4)	2 (0–9)	2 (0–8)	2 (0–6)	2 (0–4)
**Concomitant omeprazole** *n* (%)	17 (9%)	0 (0%)	4 (10%)	6 (8%)	4 (8%)	3 (30%)
**Concomitant oral contraceptives** *n* (%)	19 (11%)	0 (0%)	6 (15%)	9 (12%)	3 (6%)	1 (10%)
**Maximum dose (mg)** Median (range)	20 (2.5–40)	12.5 (5–20)	15 (2.5–40)	20 (5–40)	15 (5–30)	20 (10–20)


*n, number; mg, milligrams; PM, poor metabolizer; IM, intermediate metabolizer; NM, normal metabolizer; RM, rapid metabolizer; UM, ultrarapid metabolizer.*

### Side Effects

**FIGURE 1:**
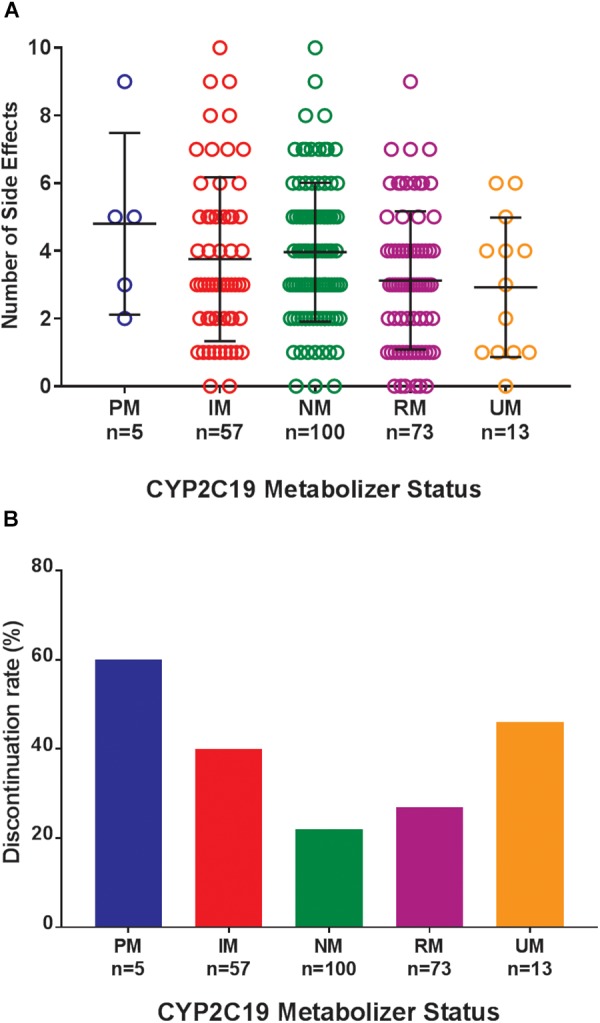
**(A)** Total number of side effects experienced during treatment with escitalopram or citalopram (es/citalopram) by 248 patients included in the tolerability analysis. CYP2C19 metabolizer status is associated with the total number of side effects experienced (*p* = 0.015). The association with CYP2C19 metabolizer status remained significant (*p* = 0.019) in a multivariate regression model that accounted for es/citalopram dose and concomitant medications. Mean and standard deviation are indicated by the bar and whiskers. **(B)** Discontinuation rates by CYP2C19 metabolizer status in the tolerability analysis with a documented reason for discontinuation of es/citalopram in the electronic medical record. PMs and IMs were significantly more likely to discontinue es/citalopram relative to NMs (*p* = 0.007, χ^2^), while RMs and UMs were not (*p* = 0.20, χ^2^). PM, poor metabolizer; IM, intermediate metabolizer; NM, normal metabolizer; RM, rapid metabolizer; UM, ultrarapid metabolizer; *n*, number.

Of the 248 inpatient pediatric patients with anxiety and/or depressive disorders assessed for tolerability, 95.6% (*n* = 237/248) had at least one side effect while prescribed es/citalopram (Table 3). CYP2C19 metabolizer status was associated with the total number of side effects experienced, with PMs having the most and UMs having the fewest side effects (Figure 1A). PMs and IMs had more total side effects compared to RMs and UMs (*p* < 0.05, Table 4). In a multivariate model, total number of side effects was positively associated with diagnosis (*p* = 0.026, Table 5) and number of concomitant psychotropic medications (*p* = 4.04 × 10^-11^), while maximum dose of es/citalopram (*p* = 0.056) and CYP2C19 metabolizer status (*p* = 0.051) were above the threshold for significance. The maximum es/citalopram dose was higher in patients with each of the side effects with the exception of nausea, suggesting the side effects may be attributed to es/citalopram (Table 4). Notably, CYP2C19 metabolizer status was not associated with maximum dose of es/citalopram (*p* = 0.3).

**Table 3:** Frequency of side effects in tolerability cohort.

	Total	PM	IM	NM	RM	UM
Side effect	*n* = 248	*n* = 5	*n* = 57	*n* = 100	*n* = 73	*n* = 13
% (*n*)		(2%)	(23%)	(40%)	(29%)	(5%)
1 or more side	96% (238)	100% (5)	96% (55)	97% (97)	92% (67)	92% (12)
effect
Activation	7% (18)	20% (1)	7% (4)	9% (9)	4% (3)	8% (1)
Drowsiness	70% (173)	80% (4)	68% (39)	73% (73)	67% (49)	62% (8)
Irritability	69% (172)	100% (5)	68% (39)	73% (73)	66% (48)	54% (7)
Insomnia	64% (158)	80% (4)	63% (36)	71% (71)	53% (39)	62% (8)
Impulsivity	40% (98)	80% (4)	42% (24)	42% (42)	33% (24)	31% (4)
Hyperactivity	48% (118)	20% (1)	12% (7)	16% (16)	10% (7)	15% (2)
Headache	36% (89)	20% (1)	44% (25)	36% (36)	32% (23)	31% (4)
Nausea	28% (69)	40% (2)	28% (16)	33% (33)	21% (15)	23% (3)
GI Toxicity	23% (58)	0	25% (14)	21% (21)	18% (13)	8% (1)
Weight Gain	17% (41)	40% (2)	18% (10)	22% (22)	10% (7)	0


*n, number; PM, poor metabolizer; IM, intermediate metabolizer; NM, normal metabolizer; RM, rapid metabolizer; UM, ultrarapid metabolizer; GI, gastrointestinal.*

**Table 4:** Associations of clinical variables with outcomes.

	Age	Race	Sex	diagnosis	CYP2C19	Adherence	*N* meds	Max dose	Cont	Omep
Sum of side effects				0.03	0.0496	0.014	3.30E-18	1.50E-08		
Activation (sum of irritability, insomnia, hyperactivity, impulsivity)					0.019	0.037	3.26E-11	5.20E-07		
Drowsiness			0.04	0.001			1.40E-05	7.80E-05		
Irritability							5.60E-05	0.004		
Insomnia				0.04			0.0001	5.10E-06		
Impulsivity					0.04	0.045	0.002	0.03		
Hyperactivity							2.10E-06	0.003		
Headache							1.10E-05	0.04		
Nausea				0.03		0.008	1.10E-06		0.038	
GI Toxicity							1.20E-05	0.03		0.009
Weight Gain		0.04			0.02		4.10E-05	0.0008		
Hospitalization days		0.04		0.01			1.50E-13	4.20E-07		
Response				0.01	0.046		0.026	0.006		
Response dose	0.03			0.003			7.00E-04	NA	0.008	


*N, number. Meds, psychotropic medications. Max, maximum. Cont, concomitant oral contraceptives. Omep, concomitant omeprazole. GI, gastrointestinal.*

**Table 5:** Multivariate model of total number of side effects.

Variable	Beta	*p*-value	*R*^2^ adj.
CYP2C19	-1.212	0.051	0.022
Contraceptives	0.285	0.48	0.009
Omeprazole	0.462	0.27	0.006
*N* meds	0.488	4.04E-11	0.262
Max dose	0.034	0.056	0.119
Adherence	0.600	0.13	0.020
Diagnosis	0.919	0.026	0.013


*Adjusted R^2^ for the model with all covariates included: 0.299. *N*, number. Meds, psychotropic medications. Max, maximum.*

**FIGURE 2:**
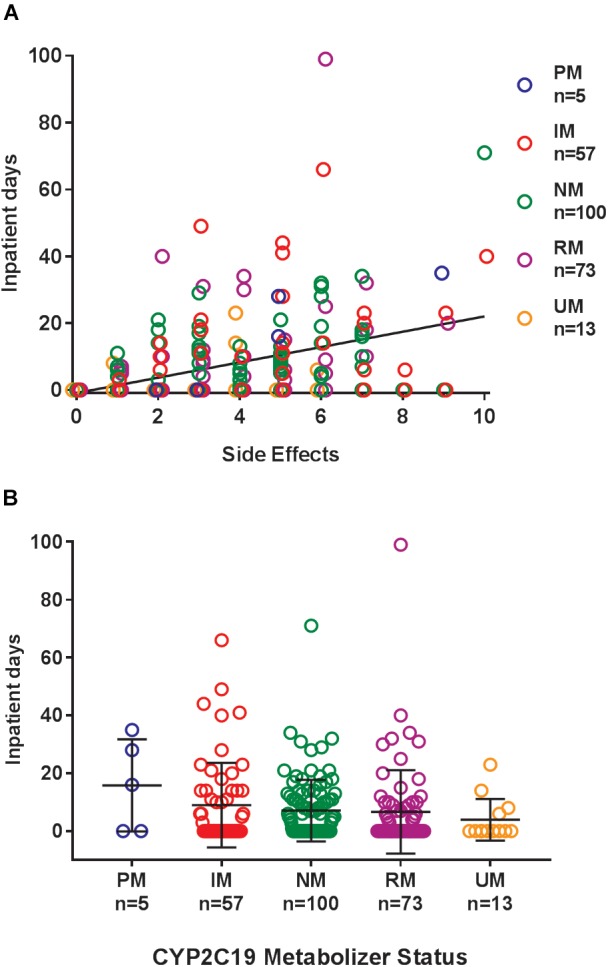
**(A)** Cumulative days in the inpatient psychiatric unit (over the entire follow-up period after the initial hospitalization) correlated with the total number of side effects in a linear model (regression line in black *p* = 2 × 10^-10^). **(B)** Cumulative days patients were admitted to the inpatient psychiatric unit during treatment with es/citalopram after the initial hospitalization by CYP2C19 metabolizer status, *p* = 0.076. Mean and standard deviation are indicated by the bar and whiskers. PM, poor metabolizer; IM, intermediate metabolizer; NM, normal metabolizer; RM, rapid metabolizer; UM, ultrarapid metabolizer; *n*, number.

Where possible, we assessed the discontinuation of es/citalopram and rehospitalization, to investigate whether these events were related to side effects. The reason for discontinuation of es/citalopram was documented in the EMR for a subset of patients (*n* = 74). PMs and IMs were significantly more likely to discontinue es/citalopram treatment than NMs (*p* = 0.007, **χ**^2^), while RMs and UMs were not (*p* = 0.20, **χ**^2^, Figure 1B). The total number of inpatient days (after the initial inpatient stay when es/citalopram was prescribed) correlated with the total number of side effects in a linear model (*p* = 2 × 10^-10^, Figure 2A), however, the CYP2C19 metabolizer status was above the threshold for significance (*p* = 0.076, Figure 2B).

**FIGURE 3:**
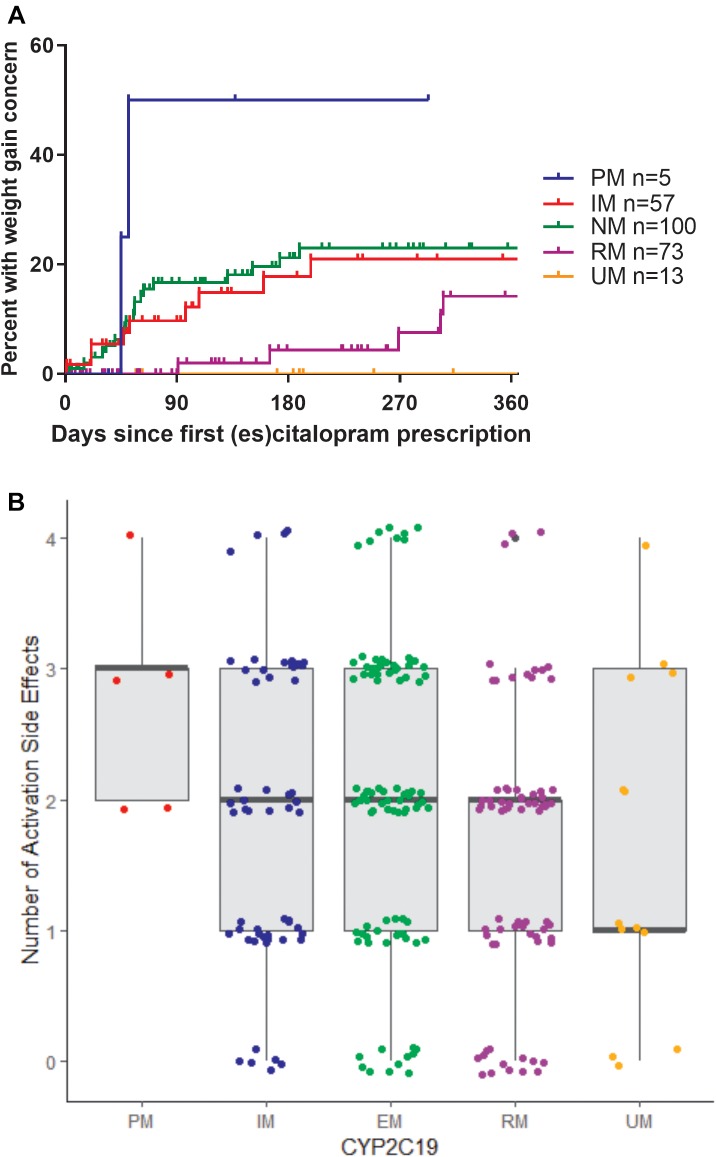
**(A)** Time to first weight gain concern during es/citalopram treatment is associated with CYP2C19 metabolizer status (*p* = 0.018, log-rank test for trend). **B**, Number of activation side effects during es/citalopram treatment is associated with CYP2C19 metabolizer status (*p* = 0.029, one-way ANOVA with test for trend). Median and interquartile range are indicated by the bar and whiskers. PM, poor metabolizer; IM, intermediate metabolizer; NM, normal metabolizer; RM, rapid metabolizer; UM, ultrarapid metabolizer; *n*, number.

In terms of individual side effects, there was a significant association between slower CYP2C19 metabolizer status and earlier weight gain (*p* = 0.018, log-rank test for trend, Figure 3A). Weight gain was also associated with race (14% in white patients, 24% in other races, 30% in black patients, *p* = 0.04 for white vs. black), maximum dose of es/citalopram (greater weight gain at higher doses, *p* = 8 × 10^-4^), and the number of concomitant psychotropic medications (*p* = 4 × 10^-5^). These factors remained significant in a multivariate regression model with the exception of maximum dose (Table 6). The number of activation symptoms a patient experienced (insomnia, irritability, hyperactivity, and impulsivity) was related to the CYP2C19 metabolizer status, with slower metabolizers experiencing more activation symptoms than faster metabolizers (*p* = 0.019, Figure 3B). The number of activation symptoms was also related to the number of concomitant psychotropic medications (*p* = 3.3 × 10^-11^) and maximum dose of es/citalopram (*p* = 5.2 × 10^-7^). All remained significant in a multivariate regression model (Table 7).

**Table 6:** Multivariate model of weight gain.

Variable	Beta	*p*-value
CYP2C19	-0.245	0.042
Contraceptives	-0.011	0.89
Omeprazole	0.107	0.18
*N* meds	0.042	0.002
Max dose	0.005	0.15
Race	-0.230	0.004


*N, number. Meds, psychotropic medications.*

**Table 7:** Multivariate model of activation side effects.

Variable	Beta	*p*-value
CYP2C19	-0.713	0.039
Contraceptives	0.109	0.63
Omeprazole	-0.124	0.59
N meds	0.178	7.40E-06
Max dose	0.023	0.02
Adherence	0.307	0.17


*N, number. Meds, psychotropic medications.*

For a subset of 26 patients in the tolerability cohort (*n* = 248), adherence concerns were noted by the clinician during the es/citalopram treatment period. Non-adherence was associated with the number of concomitant medications (more medications in the non-adherent patients, *p* = 0.015), and the total side effect burden (more side effects in the non-adherent patients, *p* = 0.014). Adherence concerns were not associated with maximum dose. Adherence concerns were associated with nausea (*p* = 0.008), with 50% of those with adherence concerns having nausea and only 25% of the rest of the tolerability cohort experiencing nausea. Fifty-seven percent of non-adherent patients had impulsivity while only 37% of the rest of the tolerability cohort experienced impulsivity (*p =* 0.045).

### Response

**FIGURE 4:**
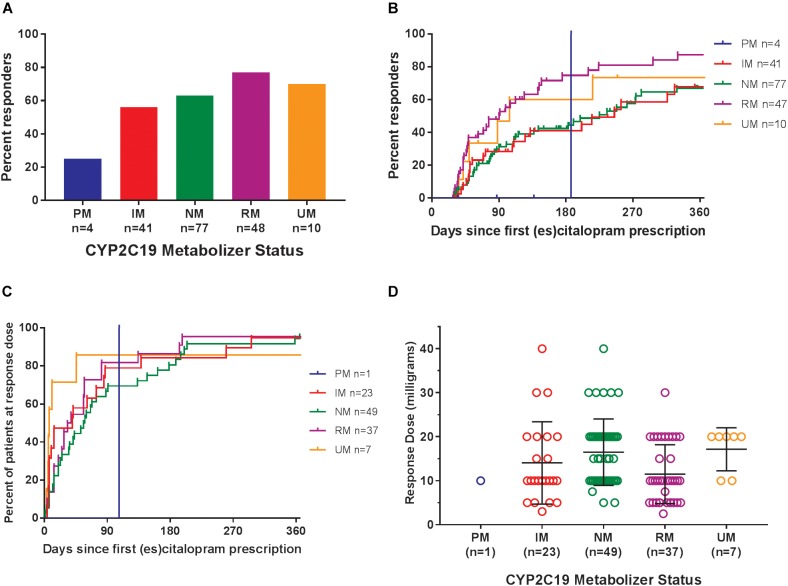
**(A)** Percentages of patients in the response analysis who achieved or did not achieve a response while prescribed es/citalopram (*p* = 0.12, χ^2^). **(B)** Time to response was associated with CYP2C19 metabolizer status (*p* = 0.005, log-rank test for trend). **(C)** Time to response dose among patients who achieved a response was not different by CYP2C19 metabolizer status (*p* = 0.27, log-rank test for trend). **(D)** Response dose was not associated with CYP2C19 metabolizer status (*p* = 0.67, one-way ANOVA with test for trend). Mean and standard deviation are indicated by the bar and whiskers. PM, poor metabolizer; IM, intermediate metabolizer; NM, normal metabolizer; RM, rapid metabolizer; UM, ultrarapid metabolizer; *n*, number.

Of the 180 pediatric patients with anxiety and/or depressive disorders assessed for response, 65% responded (achieved a Clinical Global Impression-Improvement [CGI-I] score (Guy, 1976) of 1 or 2 while prescribed es/citalopram). CYP2C19 metabolizer phenotype did not influence the proportion of patients that responded (*p* = 0.12, χ^2^, Figure 4A). However, RMs and UMs responded more quickly (*p* = 0.005, log-rank test for trend, Figure 4B) but were not prescribed their response dose earlier than other CYP2C19 metabolizer groups (*p* = 0.27, log-rank test for trend, Figure 4C). There was no association between CYP2C19 metabolizer status and response dose (*p* = 0.67, one-way ANOVA with test for trend, Figure 4D). In a multivariate regression model including CYP2C19 metabolizer status, the maximum dose of escitalopram, number of concomitant neuropsychiatric medications, omeprazole, oral contraceptives, and diagnosis, CYP2C19 metabolizer status and diagnosis were associated with response (Table 8).

**Table 8:** Multivariate model of response.

Variable	Beta	*p*-value
CYP2C19	0.385	0.03
Contraceptives	0.204	0.074
Omeprazole	-0.107	0.37
*N* meds	0.022	0.29
Max dose	0.009	0.11
Diagnosis	-0.333	0.006


*N, number. Meds, psychotropic medications.*

## Discussion

This is the first study to examine CYP2C19 metabolizer status and es/citalopram treatment outcome in children and adolescents with anxiety and depressive disorders. CYP2C19 metabolizer status explained a portion of the variability in es/citalopram response and tolerability. Response rates were similar to those observed in clinical trials of es/citalopram, but the frequency of side effects was higher than reported in prospective clinical trials, likely due to the difference in capturing of side effects and length of follow-up (Wagner et al., 2004, 2006; Goodman et al., 2005; Isolan et al., 2007; Emslie et al., 2009; Cipriani et al., 2016; Locher et al., 2017; Strawn et al., 2018). Our data suggest slower CYP2C19 metabolizers had decreased tolerability, leading to an increased risk of additional psychiatric inpatient days and discontinuation, compared to faster metabolizers. As expected, slower CYP2C19 metabolizers experienced more side effects during es/citalopram treatment, compared to faster CYP2C19 metabolizers, even after adjusting for concomitant psychotropic medications. This finding is consistent with prior work in adults (Mrazek et al., 2011; Hodgson et al., 2015; Jukic et al., 2018). The total number of side effects experienced during treatment was positively associated with higher prescribed dosages of es/citalopram after adjusting for CYP2C19 metabolizer status, indicating tailored dosing may be beneficial.

Our work revealed that the total number of side effects was associated with the number of days that patients were subsequently re-hospitalized (during es/citalopram treatment). While we lack healthcare cost data for our patients, the time spent re-hospitalized suggests higher healthcare costs for slower CYP2C19 metabolizers when standardized dosing approaches are used without consideration of CYP2C19 metabolizer status. A previous study predicted pharmacogenetic testing for major depressive disorder in adults would yield cost savings in real-world clinical settings (Maciel et al., 2018). Additionally, cost of medications was more than $1000 lower in a cohort that received pharmacogenetic testing than those that did not (Winner and Dechairo, 2015). We speculate that the decreased es/citalopram tolerability in our slower metabolizers slowed es/citalopram-related improvement. Meanwhile, slower metabolizers were more likely to discontinue es/citalopram due to side effects and/or a lack of efficacy compared to NMs, replicating findings in adults (Jukic et al., 2018). Two important treatment-related side effects of escitalopram are weight gain and activation. First, CYP2C19 activity may influence weight gain in this vulnerable population, a finding which may help explain variability in weight gain that has been prospectively observed in escitalopram-treated youth (Czaja et al., 2013; Calarge et al., 2017; Ramsey et al., 2018a). Second, activation, a constellation of side effects that often cause treatment discontinuation and decrease the likelihood of medication response (Bussing et al., 2013; Luft et al., 2018), was associated with CYP2C19 phenotype; more activation symptoms were reported in patients that metabolize es/citalopram more slowly, even after controlling for concomitant psychotropics. Taken together, these data suggest that side effects that occur during es/citalopram treatment have substantial clinical consequences, including increased re-hospitalizations and medication discontinuation, which could be reduced if metabolizer-guided dosing had been implemented.

Our treatment response findings warrant additional discussion. There was no association between metabolizer status and documented clinical improvement, but slower CYP2C19 metabolizers required significantly longer to respond. This was not completely unexpected, as studies in adults have yielded mixed conclusions on the association between CYP2C19 metabolizer status and response outcomes in adults taking es/citalopram (Altar et al., 2013; Hodgson et al., 2014, 2015), likely reflecting the complex nature of treatment response in the psychiatric setting. Defying our expectations, slower metabolizers responded more slowly than the faster metabolizers, opposite the effect seen in children with autism prescribed escitalopram (Bishop et al., 2015). The trend in our patients does not appear to be the result of physicians varying their prescribing behavior based on CYP2C19 metabolizer status, as RMs and UMs were neither prescribed the response dose more quickly nor were they prescribed a higher maximum dose of es/citalopram. The lack of relationship between CYP2C19 metabolizer status and dosing is not surprising considering the clinicians receive no tailored dosing recommendations for es/citalopram based on CYP2C19 metabolizer status. Taken together, the results highlight the potential of personalized dosing recommendations based on CYP2C19 metabolizer status to enhance treatment outcomes in youth with anxiety and/or depressive disorders.

Our study adds to a growing body of research demonstrating the feasibility of using retrospective EMR data for pharmacogenetic research in the inpatient psychiatric setting (Prows et al., 2009; Jukic et al., 2018). As EMR data extraction becomes increasingly automated, we offer a model for investigating relationships between pharmacogenetics and treatment outcomes using retrospective EMR data in the psychiatric setting.

While we present a relatively large study evaluating treatment for pediatric anxiety and depression, the number of patients that were PMs was small. The retrospective design was a limitation, and given the high degree of variability in treatment outcomes in the patients, our study was underpowered. Larger, prospective studies are needed to confirm the association of CYP2C19 metabolizer status with treatment outcomes. Additionally, concomitant medications, substance use, or symptoms of the underlying disorder being treated may have degraded our ability to causally link specific symptoms/side effects with es/citalopram treatment.

One of the strengths of our study was its real-world patient population; however, our results may not be generalizable to pediatric patients treated only in an outpatient setting because inpatients with anxiety and/or depressive disorders may have a more severe disease pathology and/or environmental stressors with distinct treatment needs. Serum concentrations of es/citalopram, which have been related to response in pediatric patients (Sakolsky et al., 2011), are not measured routinely at our institution, and therefore were not available for analysis as has recently been performed in adults (Jukic et al., 2018).

Rare *CYP2C19* variants were not included in our genotyping test; thus, in rare cases, an assigned “wild-type” (^∗^1) allele may harbor a variant with no, decreased, or increased function. An individual’s CYP2C19 metabolizing activity may also depend on other factors that we didn’t assess, including epigenetics (Helsby and Burns, 2012), comorbidities, or diet, although we did attempt to account for concomitant medications metabolized by and/or inhibitors of CYP2C19. Since CYP2C19 metabolic activity may be regulated by the estrogen receptor alpha (Helsby and Burns, 2012), analysis of the association between CYP2C19 metabolizer status and outcomes would ideally include pubertal status, but this was not available in the EMRs in this retrospective review. Response to es/citalopram is multi-factorial (Wehry et al., 2018), and other genes have been associated with escitalopram response and side effects, including (but not limited to) *SLC6A4* (Hu et al., 2007; Ng et al., 2013; Zhu et al., 2017), *HTR2A*, *GRIK4*, and *FKBP5* (Horstmann et al., 2010).

In summary, CYP2C19 metabolizer status helped to explain the wide variability in treatment outcomes we observed in children and adolescents with anxiety and/or depressive disorders prescribed es/citalopram. Collectively, our findings suggest that dosing es/citalopram based on CYP2C19 metabolizer status could improve safety and accelerate treatment response in pediatric patients. Further research is warranted to develop personalized dosing recommendations based on CYP2C19 metabolizer status and assess their impact on treatment outcomes in pediatric patients with anxiety and/or depressive disorders.

## Author Contributions

SA, LM, LR, and JS designed and performed the research, and analyzed the data. EP performed the research and contributed to new reagents/analytical tools. CP designed the research and analyzed the data. All authors wrote the manuscript.

## Conflict of Interest Statement

JS received research support from the National Institute of Mental Health and the National Institute of Environmental and Health Science, Allergan, Edgemont Pharmaceuticals, Lundbeck, Neuronetics, and Shire. He received material support from Genesight/Assurex and provided consultation to Genesight/Assurex. He is an Associate Editor for *Current Psychiatry* and the *Journal of Child & Adolescent Psychopharmacology* and receives royalties from *UptoDate* and Springer. The remaining authors declare that the research was conducted in the absence of any commercial or financial relationships that could be construed as a potential conflict of interest.

## Footnotes

1 www.r-project.org
